# Association between expatriation and HIV awareness and knowledge among injecting drug users in Kabul, Afghanistan: A cross-sectional comparison of former refugees to those remaining during conflict

**DOI:** 10.1186/1752-1505-1-5

**Published:** 2007-03-21

**Authors:** Catherine S Todd, Abdullah MS Abed, Steffanie A Strathdee, Paul T Scott, Boulos A Botros, Naqibullah Safi, Kenneth C Earhart

**Affiliations:** 1 Division of International Health & Cross-cultural Medicine, University of California, San Diego School of Medicine, 9500 Gilman Drive, La Jolla, California 92093-0622, USA; 2 National AIDS Control Program; Ministry of Public Health; Great Massoud Circle, Kabul, Afghanistan; 3 United States Military HIV Research Program/Division of Retrovirology, Walter Reed Army Institute of Research; 1 Taft Court, Suite 250; Rockville, Maryland 20850, USA; 4 Virology Research Program, United States Naval Medical Research Unit 3; Ramses Extension Street near Abbasia Fever Hospital; Abbassia, Cairo, Egypt

## Abstract

**Background:**

Little is known about human immunodeficiency virus (HIV) awareness among Afghan injecting drug users (IDUs), many of whom initiated injecting as refugees. We explored whether differences in HIV awareness and knowledge exist between Afghan IDUs who were refugees compared to those never having left Afghanistan.

**Methods:**

A convenience sample of IDUs in Kabul, Afghanistan was recruited into a cross-sectional study through street outreach over a one year period beginning in 2005. Participants completed an interviewer-administered questionnaire and underwent voluntary counseling and testing for HIV, syphilis, hepatitis B surface antigen, and hepatitis C antibody. Differences in HIV awareness and specific HIV knowledge between IDU who lived outside the country in the last decade versus those who had not were assessed with logistic regression.

**Results:**

Of 464 IDUs, 463 (99%) were male; median age and age at first injection were 29 and 25 years, respectively. Most (86.4%) had lived or worked outside the country in the past ten years. Awareness of HIV was reported by 46.1%; those having been outside the country in the last decade were significantly more likely to have heard of HIV (48.3% vs. 31.7%; OR = 2.00, 95% CI: 1.14 – 3.53). However, of those aware of HIV, only 38.3% could name three correct transmission routes; specific HIV knowledge was not significantly associated with residence outside the country.

**Conclusion:**

Accurate HIV knowledge among Afghan IDUs is low, though former refugees had greater HIV awareness. Reported high-risk injecting behavior was not significantly different between IDU that were refugees and those that did not leave the country, indicating that all Afghan IDU should receive targeted prevention programming.

## Background

Military conflict frequently results in the displacement of large numbers of people, often to neighboring countries. The link between spread of human immunodeficiency virus (HIV) and population migration is controversial [1-3]; however, post-conflict populations have been identified as being at particular risk of HIV acquisition due to vulnerabilities created by displacement, lack of HIV knowledge, and tendencies to engage in riskier behaviors [4-7]. International literature suggests that refugees have comparatively less knowledge about HIV and engage disproportionately in high risk behaviors compared to their counterparts who remain in the country [7-10]. However, there has been little scrutiny of whether exposure to a setting of greater knowledge of HIV and other blood-borne infections results in transferred knowledge among those who repatriate to their country of origin.

Afghanistan is currently a fragile state, still reeling from the effects of nearly thirty years of civil war and current political instability in several regions of the country [11]. While Afghanistan is considered a low HIV prevalence country, there have been few assessments of HIV knowledge [12]. Measuring the knowledge of HIV and ability to identify and access tools for prevention is pertinent for Afghanistan due to factors that may result in a concentrated epidemic among injecting drug users (IDU). Iran, Pakistan, Uzbekistan, Tajikistan, and western China are all experiencing rapid increases in HIV prevalence among IDU in multiple cities [3]. Approximately six million Afghans were displaced to these countries, particularly Iran and Pakistan, during the last three decades of conflict [13]. The number of IDUs in Afghanistan is believed to be increasing, possibly due to learned injecting behaviors from countries of refuge [14]. In Pakistan, Afghan IDU were found to have less HIV knowledge than their Pakistani counterparts,[9] but no comparison has been performed between IDU who were refugees and those not leaving the country.

The purpose of this paper is to examine whether displacement outside Afghanistan is associated with knowledge of HIV and other blood-borne infections among IDU in Kabul, Afghanistan. This information is important because it indicates the penetration of HIV awareness and harm reduction programming in both Afghanistan and in neighboring countries within this high risk and potentially marginalized group and the accuracy of messages intended for this group. This manuscript reports the association between residence outside Afghanistan and HIV awareness and knowledge and assesses the level of accurate HIV knowledge among IDUs in Kabul between 2005 and 2006.

## Methods

### Study Design and Participants

This cross-sectional study was conducted in Kabul, the capital of Afghanistan, between June, 2005 and June, 2006. All study activities occurred at the Voluntary Counseling and Testing (VCT) Center at the Central Polyclinic, an Afghan Ministry of Public Health facility, with participants referred through outreach personnel already working with the drug-using community. Eligible participants were those reporting injecting drugs within the past six months (confirmed through injection stigmata), aged 18 years or greater, and able to provide informed consent. Prior to enrollment, the research study was approved by the investigational review boards of the Afghan Ministry of Public Health; the University of California, San Diego; the United States Naval Medical Research Unit No. 3; and the Walter Reed Army Institute of Research.

### Measurement of variables and outcomes of interest

The questionnaire included questions pertaining to sociodemographics, travel, incarceration, and medical histories, and drug use and sexual behaviors. Time outside the country was first assessed with a general question: "Have you lived/worked outside Afghanistan for any period of time in the last 10 years?" Those responding affirmatively were asked to define the time period of expatriation by the government in power at the time of leaving the country: during the Russian occupation (1979–1989), during Najibullah and the mujaheddin (1989 – 1995), during the Taliban (1996 – 2001), or during the military action deposing the Taliban (2001 – 2003). Country of displacement and reason(s) for leaving were also assessed with multiple responses allowed.

The outcome of interest, awareness of HIV infection, was assessed with the question,"Have you ever heard of HIV?." Knowledge about HIV was gauged through one open-ended (e.g. "List the ways you know that HIV is spread") and twelve true/false questions which included assessment of: transmission routes (e.g."Which of the following activities can result in HIV infection: mosquito bites, sexual intercourse, hugging/kissing"), longevity and treatment for HIV (e.g. "There are medicines that can cure a person of their HIV infection"), and means of prevention (e.g."When it comes to getting HIV from sexual intercourse, using a condom is a good way to reduce your chance of getting HIV").

### Procedures

Potential participants were accompanied to the VCT Center by an outreach worker or other IDU of their acquaintance. At the center, trained study representatives matched to participant gender explained the study in a confidential setting, obtained informed consent, administered the questionnaire by interview, and performed serologic testing. Each participant was assigned a unique study number. No data was recorded on those declining or ineligible for study entry.

Pre- and post-test counseling and rapid whole blood testing for HIV, syphilis, hepatitis C antibody, and hepatitis B surface antigen testing was subsequently performed with positive tests undergoing confirmation on site. All participants received risk reduction counseling, condoms and sterile syringes, with referrals for detoxification and needle and syringe programs (NSPs) upon request.

### Statistical Analysis

The outcomes of interest for this analysis were familiarity with and correct knowledge of HIV among those IDU having left Afghanistan compared to those having not left in the last decade. Our sample size was sufficient to detect at least an 18% difference in HIV awareness or knowledge between those having left Afghanistan within the last decade and those not having left the country (power = 80, two-sided alpha = 0.05). To measure familiarity with HIV, the dichotomous variable HIV awareness was utilized with a yes or no response. Correct transmission knowledge was defined as three correct responses and no incorrect responses to reported modes of transmission. Continuous variables, such as age of initiating injection use, were transformed to dichotomous variables using the median age as the cut-off point. Univariate logistic regression was performed to identify potential differences in demographics and high risk behaviors between IDU who had left Afghanistan ever and in the last decade. Logistic regression was performed to test for associations between HIV familiarity and knowledge and time outside the country. A separate logistic regression analysis was performed to determine whether length of time outside the country, a categorical variable with time outside the country increasing with ascending number, affected the strength of association between variables related to expatriation.

## Results

A total of 464 IDU participated in this study, of whom the majority were male (n = 463) and Afghan (n = 459). Most (86.4%, n = 400) had lived or worked outside the country in the past ten years, with war (80.7%, n = 338; multiple responses permitted), work (15.5%, n = 65), political repression (1.2%, n = 5), education (0.7%, n = 3), or other factors (1.9%, n = 8) cited as reasons for leaving the country. The majority of participants moved during the Taliban regime (50.1%), with 31.9% leaving during Najibullah's leadership (1989 – 1995), 15.3% leaving during the Russian invasion, and 2.4% leaving at the time of United States-led military activity against the Taliban. Many (37.7%) had been to more than one country during periods of expatriation, with Iran being the most popular country of refuge (61.3%), followed by Pakistan (32.3%) and other countries, including India, the Arab Emirates, and Tajikistan (6.4%).

The prevalence and correlates of having left Afghanistan in the last decade are displayed in Table 1. Generally, those who resided outside Afghanistan were less likely to report using a new needle with each injection and marginally more likely to report prior condom use (Table 1). The association between using a new needle with each injection never having left Afghanistan persisted after adjustment for potential confounders of prior incarceration and monthly earnings (AOR = 0.51, 95% CI: 0.21 – 0.88). IDU who reported never having left Afghanistan (9.3%, n = 43) did not differ significantly from those who had ever left the country (data not shown).

**Table 1:** Correlates of living outside Afghanistan in the last decade among injecting drug users in Kabul (n = 463).

Variable	Non-refugee last decade, N, %	Refugee last decade, decade, N, %	O.R., 95% CI
Age (n = 463):			1.02, 0.59 – 1.76
≤ 30 years	38, (60.3%)	239, (59.8%)	
> 30 years	29, (39.7%)	161, (40.3%)	
Civil Status (n = 463):			1.06, 0.62 – 1.80
Married	32, (50.8%)	209, (52.3%)	
Educational Level (n = 463):			0.96, 0.50 – 1.86
≤ 8 years	50, (79.4%)	320, (80.0%)	
> 8 years	13, (20.6%)	80, (20.0%)	
Monthly Income (n = 463):			0.65, 0.38 – 1.11
≤ 4900 Afghanis	37, (58.7%)	275, (68.8%)	
> 4900 Afghanis	26, (46.3%)	125, (31.3%)	
Duration of Injecting (n = 463):			1.27, 0.74 – 2.18
≤ 3 years	38, (60.3%)	218, (54.5%)	
> 3 years	25, (39.7%)	182, (45.5%)	
Age Initiated Injecting (n = 463):			1.12, 0.66 – 1.92
< 26 years	36, (57.1%)	217, (54.3%)	
> 26 years	27, (42.9%)	183, (45.8%)	
Receptive Needle Sharing in Last Six Months (n = 463):			0.98, 0.55 – 1.77
Yes	18, (28.6%)	113, (28.3%)	
Distributive Needle Sharing in the Last Six Months (n = 463):			1.02, 0.57 – 1.84
Yes	18, (28.6%)	116, (29.0%)	
**Always Use New Needle With Each Injection (n = 462):**			**0.49, 0.29 – 0.85**
**Yes**	**27, (42.9%)**	**108, (27.1%)**	
Ever Use Condoms (n = 431):			2.28, 0.94 – 5.50
Yes	6, (10.5%)	79, (26.8%)	
Ever Incarcerated (n = 463):			1.00, 0.59 – 1.72
Yes:	36, (57.1%)	229, (57.1%)	
Inject Drugs in Jail (n = 265):			1.26, 0.42 – 2.09
Yes	10, (31.3%)	66, (29.9%)	

Awareness of HIV was reported by 46.1% (n = 214) of participants; IDU having been outside the country in the last decade were significantly more likely to have heard of HIV (48.3% vs. 31.7%; OR = 2.00, 95% CI: 1.14 – 3.53). This relationship remained significant when analyzed with potential confounders of educational level, prior incarceration, and monthly income level (AOR = 2.08, 95% CI: 1.16 – 3.72). Country of refuge was not significantly associated with HIV awareness; however, duration of expatriation was marginally positively associated with HIV awareness (OR = 1.27, 95% CI: 0.98 – 1.65).

Among those aware of HIV, residence outside the country in the last decade did not affect the source of information about HIV (friends, p = 0.89; TV/print media, p = 0.34; medical personnel, p = 0.66; or addiction treatment centers, p = 0.27). Of those aware of HIV, 95.3% (n = 204) claimed knowledge of routes of transmission. However, when asked to name transmission routes, only 38.3% of those aware of HIV were able to provide three correct and no incorrect responses, which was not associated with having lived outside the country in the last decade (p = 0.89; OR = 0.93, 95% CI: 0.36 – 2.39). Duration spent outside the country was inversely associated with ability to name three correct transmission routes (OR = 0.64, 95% CI: 0.43 – 0.95).

No significant differences in specific HIV knowledge content areas emerged between IDUs living outside Afghanistan in the last decade and IDUs who remained within the country; however, the low numbers in each group reduced statistical power considerably (Table 2). For participants who had ever resided outside of Afghanistan, the level of specific HIV knowledge was assessed according to the duration of time spent outside the country. Knowledge that using new needles with each injection reduce HIV risk, that HIV is infectious forever, or, marginally, that HIV may be transmitted by donated blood was positively related to length of time outside the country, while knowing that HIV is not transmitted by hugging/kissing, mosquito bites, or lack of hygiene was inversely related to time outside the country (Table 3).

**Table 2:** Differences in correct HIV knowledge among injecting drug users (IDUs) in Kabul, Afghanistan (n = 214).

Question Topic	Overall	Live in Afg. > 10 yrs n,(%)	Lived Outside Afghanistan n,(%)	OR, 95% CI
HIV infectious forever:				0.66, 0.26 – 1.66
	97, (45.5%)	11, (55.0%)	86, (44.6%)	
HIV can not be detected just by looking at a person				1.43, 0.55 – 3.74
	91, (42.7%)	7, (35.0%)	84, (43.5%)	
Medicines cannot cure HIV				0.86, 0.33 – 2.26
	68, (31.9%)	7, (35.0%)	61, (31.6%)	
Used needles transmit HIV				0.60, 0.17 – 2.13
	166, (71.9%)	17, (85.0%)	149, (77.2%)	
HIV transmitted by donated blood				0.68, 0.27 – 1.70
	87, (41.2%)	10, (50.0%)	77, (58.9%)	
HIV not transmitted by mosquito bites:				1.20, 0.48 – 3.00
	114, (54.0%)	10, (50.0%)	104, (54.5%)	
HIV not transmitted by lack of hygiene:				1.70, 0.67 – 4.35
	109, (51.9%)	8, (40.0%)	101, (53.2%)	
HIV transmitted sexually				1.58, 0.57 – 4.36
	165, (77.8%)	14, (70.0%)	151, (78.6%)	
HIV not transmitted by hugging/kissing				1.15, 0.45 – 2.90
	122, (58.1%)	11, (55.0%)	111, (58.4%)	
Condoms reduce risk of sexual transmission of HIV				1.92, 0.76 – 4.86
	127, (59.6%)	9, (45.0%)	118, (61.1%)	
Using a new needle with each injection reduces HIV risk				0.57, 0.22 – 1.46
	101, (47.4%)	12, (60.0%)	89, (46.1%)	

**Table 3:** Specific HIV knowledge among injection drug users in Kabul, Afghanistan.

Question Topic	Correct Knowledge %, (n)	OR, 95% CI
**HIV infectious forever (n = 196):**		**1.47, 1.00 – 2.15**
Left During Taliban/U.S. Invasion	37, 38.9%	
Left During Najibullah/Mujaheddin	31, 46.3%	
Left During Russian Occupation	20, 58.8%	
HIV can not be detected just by looking at a person (n = 196):		1.20, 0.82 – 1.75
Left During Taliban/U.S. Invasion	38, 40.0%	
Left During Najibullah/Mujaheddin	28, 41.8%	
Left During Russian Occupation	17, 50.0%	
Medicines cannot cure HIV (n = 196):		0.84, 0.56 – 1.27
Left During Taliban/U.S. Invasion	33, 34.7%	
Left During Najibullah/Mujaheddin	18, 26.9%	
Left During Russian Occupation	10, 29.4%	
Used needles transmit HIV (n = 196):		0.92, 0.59 – 1.43
Left During Taliban/U.S. Invasion	77, 81.1%	
Left During Najibullah/Mujaheddin	47, 70.1%	
Left During Russian Occupation	28, 82.4%	
HIV transmitted by donated blood (n = 196):		1.44, 0.98 – 2.11
Left During Taliban/U.S. Invasion	34, 36.2%	
Left During Najibullah/Mujaheddin	27, 40.3%	
Left During Russian Occupation	19, 55.9%	
**HIV not transmitted by mosquito bites (n = 196):**		**0.65, 0.44 – 0.95**
Left During Taliban/U.S. Invasion	60, 63.8%	
Left During Najibullah/Mujaheddin	29, 43.3%	
Left During Russian Occupation	16, 47.1%	
**HIV not transmitted by lack of hygiene (n = 196):**		**0.46, 0.31 – 0.69**
Left During Taliban/U.S. Invasion	63, 67.7%	
Left During Najibullah/Mujaheddin	27, 40.3%	
Left During Russian Occupation	12, 35.3%	
HIV transmitted sexually (n = 196):		0.84, 0.54 – 1.31
Left During Taliban/U.S. Invasion	77, 81.1%	
Left During Najibullah/Mujaheddin	50, 74.6%	
Left During Russian Occupation	26, 76.5%	
**HIV not transmitted by hugging/kissing (n = 196):**		**0.57, 0.38 – 0.84**
Left During Taliban/U.S. Invasion	64, 68.8%	
Left During Najibullah/Mujaheddin	33, 49.3%	
Left During Russian Occupation	15, 44.1%	
Condoms reduce risk of sexual transmission of HIV (n = 196):		0.84, 0.57 – 1.23
Left During Taliban/U.S. Invasion	62, 65.3%	
Left During Najibullah/Mujaheddin	38, 56.7%	
Left During Russian Occupation	20, 58.8%	
**Using a new needle with each injection reduces HIV risk (n = 196):**		**1.58, 1.08 – 2.31**
Left During Taliban/U.S. Invasion	37, 38.9%	
Left During Najibullah/Mujaheddin	33, 49.3%	
Left During Russian Occupation	21, 61.8%	

## Discussion

In 2005, Afghans represented the largest group of refugees globally with an estimated remaining total of 1.9 million, despite the repatriation of an estimated three million people between 2002 and 2004 [15-17]. While HIV awareness among Afghan refugee drug users living in Pakistan has been previously assessed,[9] this is the first study to our knowledge to examine potential differences in HIV knowledge between repatriated Afghans and those who did not leave the country. We detected a statistically significant difference in levels of HIV awareness between those IDU having lived outside the country and those not leaving Afghanistan; however, correct HIV knowledge was low and risky injecting practices were common for both groups, foreshadowing a public health catastrophe should HIV transmission accelerate within this population.

Nearly half of IDU surveyed in Kabul were aware of HIV, consistent with the 57% of IDU aware of HIV surveyed in Kabul and Herat in 2004–2005 by Action Aid.(personal communication, Dr. J. Foran) Awareness of HIV among IDU in this study was considerably higher than the 4.3% measured among Afghan drug-using refugees residing in Quetta in 2001 [9]. In a study assessing HIV and hepatitis C prevalence and correlates among drug users in Pakistan in 2003, 63.2% of Afghan drug users surveyed in Quetta were aware of HIV compared to 82.8% of Pakistani drug users (p < 0.001).(Personal communication, Dr. I. Kuo) This interval change for both Afghan and Pakistani drug users in two years may reflect greater emphasis on programming for drug users in Pakistan, perhaps in response to HIV outbreaks among IDU in several Pakistani cities detected during that time period [3]. These data must be interpreted with caution as both studies were cross-sectional and involve different individuals.

The greater HIV awareness among those who had lived or worked outside Afghanistan in the last decade may be attributed to increased public and IDU-focused education efforts in Iran and Pakistan, the countries which provided refuge to the majority of Afghans during the wars and currently experiencing concentrated HIV epidemics among IDU in several sites [3,18]. Longer duration of time outside the country was marginally associated with HIV awareness, possibly reflecting integration of long-term refugees with the host society leading to increased access to health care and HIV information. The lack of access to health services and differences in language, which may be overcome with acculturation during a longer duration of residence, were noted to be barriers to HIV knowledge acquisition in recent studies among African refugees residing in the United States and Denmark [10,19].

There was some parity between knowledge and behavior as IDU living outside Afghanistan in the last decade were more likely to have used a condom; however, they were less likely to use new syringes with each injection. In Afghanistan and Iran, disposable syringes may be purchased from pharmacies legally and at low cost [20]. However, in Pakistan, repackaging, sale, and use of previously-used syringes is attributed to high prevalence of hepatitis B and C [21,22]. The widespread knowledge of this risk may prevent IDUs from purchasing syringes but instead promote sharing by obtaining syringes from within their social network [23]. Alternatively, harm reduction programming within Afghanistan may have accessed more IDU or better communicated the need to use new needles with each injection. Though this comparison did not reach statistical significance, IDU not having left Afghanistan more frequently stated that using a new needle/syringe with each injection would reduce risk of HIV than those who lived in other countries within the last decade.

Awareness of HIV did not necessarily reflect accurate knowledge about HIV and many IDU were unable to supply correct responses to specific knowledge questions. While it is reassuring that most IDU correctly identified used needles and sexual intercourse as routes of infection in true/false questions, it is more concerning that less than 60% were able to definitively state that using a new needle with each injection or using a condom could reduce the risk of HIV transmission. Additionally, those living outside the country in the last decade were not more likely to have knowledge about modalities of HIV prevention, indicating that prevention and education efforts, both in Afghanistan and neighboring countries, are not reaching the most vulnerable populations.

This study has several limitations that must be considered. First, due to convenience sampling, the results may not be generalizable to all IDU in Kabul. Next, the interview format may have resulted in socially-desirable response on HIV awareness and reported high risk behaviors. We attempted to minimize this by having an interviewer of the same gender and administering the questionnaire prior to pre-test counseling. Last, as many participants sought refuge in multiple countries, we cannot definitively ascertain degree of knowledge imparted in a specific setting. This applies not only to countries of refuge but the possibility that this information was accessed upon return to Afghanistan. However, we believe this is unlikely due to the small number of programs focusing on HIV education for IDU and other groups in Kabul at the time of the study.

## Conclusion

While prevalence of HIV within Afghanistan is currently low, the low level of knowledge and high level of risky behaviors among IDU may precipitate a concentrated epidemic with devastating public health consequences in this post-conflict setting [3]. Although HIV awareness among returning refugees appears to be higher, neither specific HIV knowledge nor many high risk behaviors (e.g. injecting in prison, receptive or distributive needle sharing in the last six months) were significantly different from those IDU who had not left Afghanistan in the last decade. HIV prevention programs have been demonstrated to improve HIV knowledge and reduce high risk sexual behavior among high risk groups in Sierra Leone, a post-conflict setting [24]. HIV prevention programs are urgently needed in Afghanistan, however, these programs must be culturally appropriate. Evidence supports the efficacy and cultural acceptability of needle and syringe programs and opioid substitution therapy in countries in the South and Central Asian region, but there are no reports on the implementation and efficacy of harm reduction programs in post-conflict settings [25-27]. Returning refugees should receive special attention as injecting is believed to be an imported behavior and returning refugees engage in high-risk injecting practices despite HIV awareness, potentially influencing drug users that never left Afghanistan [14]. Countries in which large numbers of Afghan refugees reside should also increase efforts to reach these vulnerable populations with HIV prevention efforts.

## Abbreviations used

HIV = human immunodeficiency virus

IDU = injecting drug user

NSP = needle and syringe program

VCT = voluntary counseling and testing

## Conflict of Interests

All authors declare that they have no financial or non-financial competing interests related to this manuscript.

## Authors' contributions

CT participated in protocol design and study implementation and performed data analysis and manuscript preparation; AA supervised study conduct and data entry; SS developed the questionnaire and participated in manuscript preparation; PS participated in study implementation and manuscript preparation; NS participated in protocol development, and study implementation; BB assisted with project implementation and study conduct; and KE developed the protocol and participated in manuscript preparation.
